# Effect of Resin and Blocked/Unblocked Hardener Mixture on the Production of Epoxy Foams with CO_2_ Blocked Hardener in Batch Foaming Process

**DOI:** 10.3390/polym11050793

**Published:** 2019-05-02

**Authors:** Christian Bethke, Sandra A. Sanchez-Vazquez, Daniel Raps, Gökhan Bakis, Simon Bard, Uy Lan Du Ngoc, Altstädt Volker

**Affiliations:** 1 Department of Polymer Engineering, University of Bayreuth, Universitaetsstrasse 30, 95447 Bayreuth, Germany; christian.bethke@uni-bayreuth.de (C.B.); salinesv@gmail.com (S.A.S.-V.); daniel.raps@gmx.net (D.R.); goekhan.bakis@basf.com (G.B.); simon.bard@uni-bayreuth.de (S.B.); 2 School of Materials Engineering, University Malaysia Perlis, Kompleks Pusat Pengajian Jejawi II, Arau 02600, Perlis, Malaysia; uylan@unimap.edu.my; 3 Bavarian Polymer Institute and Bayreuth Institute of Macromolecular Research, University of Bayreuth, Universitaetsstrasse 30, 95447 Bayreuth, Germany

**Keywords:** epoxy foam, carbamate, CO_2_ foaming, epoxy resin, batch foaming, structural foam

## Abstract

The present study focuses on the processing and properties of epoxy foams by the use of CO_2_ blocked hardener N-aminoethylpiperazine (B-AEP) and different resins. Although some studies described the foaming with carbamates, little attention has been given to the interaction of resin properties (such as viscosity) on the foaming performance. Therefore, two resins, diglycidyl ether of bisphenol-A (DGEBA) and epoxy novolac (EN), as well as their 50:50 blend, were foamed with B-AEP and unblocked/blocked AEP hardener mixtures in a batch foaming process. Furthermore, the commercially available chemical blowing agent para-toluenesulfonyl hydrazide (TSH) was used as a benchmark for commonly used chemical blowing agents. The lowest density in this study was reached by the DGEBA+B-AEP system in the range of 215 kg/m^3^ with the drawback of an inhomogeneous cell structure and high cell size distribution. The best cell morphology and lowest cell size distribution was reached with the EN+15:85% unblocked:blocked hardener mixture, resulting in a density in the range of 394 kg/m^3^. A syntactic foam was achieved by a DGEBA+50:50% unblocked:blocked hardener mixture with a density of around 496 kg/m^3^. It was found that a higher viscosity of the resin lead to an increase in the density and a decrease in the cell size distribution range as a result of a closer expansion time window.

## 1. Introduction

Epoxy foams have been known since the early 1950s [1]. Their main advantages are their thermosetting molecular structure, leading to a high degree of crosslinking and high molecular weight. This provides outstanding characteristics in thermal stability, chemical resistance, and electrical properties [2,3]. Furthermore, they possess adhesive properties toward other materials, excellent rigidity, moisture resistance, and low shrinkage [4]. Due to these specific characteristics, epoxy foams are mainly applied where the performance of thermoplastic foams is not sufficient [5]. Consequently, their main applications are found in electronics, marine, aeronautics, and space components [3,5,6,7]. Present investigations focus on an efficient production and use of epoxy-based shape memory foams for the construction, transportation, and textile industries [8].

One of the main challenges for the application of epoxy foaming is the timing of blowing and hardening. Depending on the resin-hardener system, the curing can take place either at room temperature or at elevated temperatures. This requires well-defined systems where the decomposition temperature of the blowing agent matches with the optimum temperature of the curing agent, in order to obtain a proper foam quality [3,4]. The precuring of the resin systems for the adjustment of viscosity was found to be beneficial for foam morphology in the studies of Takiguchi [3] and Lyu [9]. The addition of short fibers to a foaming epoxy system was also found to lead to more homogeneous cell morphology, next to an increase of mechanical performance [10]. Alternatively, syntactic foams, where several types of hollow particles or foam particles with a defined size are mixed into the resin hardener system, are used for weight reduction and cushioning purposes [11,12,13,14].

In theory, every epoxy resin system can be foamed. However, due to processability, end use properties, and costs, there is only a small number of well-established systems that fulfill all requirements. The most promising and practical used resins for foaming applications, in research and industry, are (i) epoxy-novolac (EN)-based resins with different molecular weights and functionalities (containing three to six epoxy groups per molecule), (ii) the two functional diglycidyl ether of bisphenol-A (DGEBA) resin, and (iii) the tetrafunctional tetraglycidyl-tetrahydroxyphenolate (TGTHP) resin [2,3,4,15,16,17]. The most useful blend systems are mixtures of the low-viscosity DGEBA and higher viscosity novolacs or N-glycidylamines, due to the broad range of possible viscosities [6]. As for the curing process, different types of hardeners can be used. The hardener is chosen by its reactivity, molecular structure, and corrosivity [6,15].

However, a disadvantage for epoxy foams is that many of the established organic foaming agents and/or their obtained chemical by-products are toxic, harmful, or explosive, and consequently have gained negative reputations within the last years in regards to environment and health safety [16]. Projects such as the “Significantly New Alternatives Policy” program in northern America leads to the evaluation of hazardous chemicals for health and the environment and its smooth transition to safer alternatives. Within this program, a high number of foam blowing agents are listed [18].

One possibility to overcome the problematic toxic substances and provide alternatives to support this regulation is the use of CO_2_ as a harmless blowing agent. Gaseous or supercritical CO_2_ as blowing agents are known to work well with thermoplastics; however, both types of CO_2_ are not easy to combine with established ways of thermoset processing [19]. To bypass the fast diffusion of gaseous CO_2_ out of the curing systems, carbamate salts are a promising approach to introduce CO_2_ during the foaming process as a chemical blowing agent. Therefore, the amine hardener is blocked with CO_2_ in order to obtain its carbamate salt structure. At elevated temperatures, the carbamate decomposes releasing the CO_2_ and the respective amine, leading to a simultaneous curing and blowing process [4,7,19]. Thus, the stability of the mixed resin-hardener system should be increased compared to systems containing chemical blowing agents and amine hardeners only, as the carbamate is not able to cure the resin and does not decompose until a certain temperature [4,19].

The use of carbamates for foaming epoxy resins was firstly described in the 1960s [7]. However, this foaming method did not gain much attention in science due to the broad diversity of commonly available chemical blowing agents (CBA) in the past. With upcoming regulations of common CBAs, this foaming method has been rediscovered in science and gains more interest due to its environmental-friendly approach [4,19]. An additional positive side effect of the carbamate salts is that the whole system is fully involved in the foam, without side products left.

The ability to use CO_2_ as a latent blowing agent through blocking hardeners has recently been reviewed by Ren et al. For foaming, the resin system DGEBA with B-AEP, fumed silica (nucleating agent), and PEG-b–PPG-b–PEG (surfactant) was used. They achieved low densities (~300 kg/cm^3^) and good thermal stability (above 300 °C) [4]. Next to the commercially established N–aminoethylpiperazine (AEP) liquid hardener, his research also investigates other amine hardeners, such as m-xylenediamine and 4–4′diaminodicyclohexylmethane for the formation of carbamate salts [19]. The results show that the carbamate salts can be presented as powders or gel-like salts, depending on the molecular structure. With regards to the processability, gel-like carbamates are less suitable. Their studies also showed that an acid dissociation constant (pKa) value higher than nine is required in order to achieve a proper blocking CO_2_ reaction at ambient conditions [19]. The decomposition at elevated temperatures lead to the expectation of a long pot life of up to 180 days, which has been stated by Ren et al. [4]. 

To the authors’ best knowledge, very few publications can be found that address the issue of foaming epoxy resins with a CO_2_ blocked hardener. An industrial application with such systems was found in special gluing and adhesive applications [20,21]. Thus, next to evaluating various amine hardeners for blocking reaction and precuring, the dependence of the foaming behavior on different epoxy resins and its viscosity is also of interest for the exploitation of advanced applications. The aim of this study is to investigate the basic foaming procedure of thermoset systems with the CO_2_ blocked hardener B-AEP, the use of a hardener mixture of blocked and unblocked hardener, as well as the influence of the resin matrix on the foam morphology.

## 2. Materials and Methods 

### 2.1. Materials

For the synthesis of the blocked hardener, gaseous bottled CO_2_ with a quality grade of 4.5 (Rießner Gase, Lichtenfels, Germany) was used. Technical-grade ethanol with a purity of 98.9% denatured with 1.1% butanone (CSC Jäckle Chemie GmbH & Co. KG, Nuernberg, Germany) was used as received. The hardener, N-aminoethylpiperazine (AEP) with a *M_w_* of 129 and an amino hydrogen equivalent weight (AHEW) of 43.1 g/mol (Sigma Aldrich, St. Louis, MO, USA) was used as received. When the AEP is blocked with CO_2_, the B-AEP owns a *M_w_* of 190.8 g/mol and an AHEW of 63.6 g/mol [4]. The B-AEP was synthesized and characterized according to literature [4] and [19] with slight adaptations to own equipment. For details on synthesis, see Appendix A. The detailed synthesis is described in the additional information sheet. The used resins were (i) diglycidylether of bisphenol-A (DGEBA) supplied as Baxxores 2200 with an average *M_w_* of 340 g/mol, an average epoxy equivalent weight (EEW) of 182 g/mol, and a viscosity of 8000 to 10,000 mPa·s at 25 °C [22] (BASF SE, Ludwigshafen, Germany) and (ii) the epoxy-based Novolac D.E.N 438 with an average calculated *M_w_* of 650 g/mol, an average EEW of 179 g/mol, and a viscosity of 31,000 to 40,000 mPa·s at 51.7 °C [23] (Dow Chemicals, Stade, Germany). As a benchmark, the chemical blowing agent para-toluenesulfonyl hydrazide (TSH) releasing N_2_ and H_2_O [24] supplied as Tracel TSH 75 K1P (Tramaco, Tornesch, Germany) was used. For batch foaming, a self-made aluminum mold with a geometry of 30 mm × 30 mm × 10 mm (l × w × h) was used. Loctite Frerotte 770-NC (Henkel, Duesseldorf, Germany) was used as the mold-releasing agent.

### 2.2. Procedures

#### 2.2.1. Blending of Resins and Hardeners

Firstly, DGEBA and EN were pre-heated at 90 °C for 30 min, in order to decrease the viscosity. Subsequently, the resins were mixed in a glass vessel fixed inside a heated oil bath. Mechanical stirring was carried out for 10 min at 80 °C for all blends with an overhead mixer (IKA, Staufen im Breisgau, Germany). The epoxy equivalent weight (*EEW*) of a resin blend and the amino hydrogen equivalent weight (*AHEW*) of a hardener mixture were calculated as shown in Equation (1):

(1) $$EW_{X}=\frac{1}{\left(\left(\frac{Ratio_{X1}}{EW_{X1}}\right)+\left(\frac{Ratio_{X2}}{EEW_{X2}}\right)\right)}$$

The calculated equivalent weight of blend or mixture (*EW_X_*) was further used to prepare the stoichiometric foaming systems as shown by Equation (2) for the resin amount:

(2) $$m_{Resin}=m_{Total}\cdot \frac{EEW_{Resin}}{\left(EEW_{Resin}+AHEW_{Hardener}\right)}$$

The required amount of hardener was calculated based on the resin (*m_Resin_*) and total (*m_Total_*) amount. 

#### 2.2.2. Foaming

When using epoxy novolac, the mold and resin was pre-heated to 80 °C, while for the DGEBA/EN blend systems, pre-heating to 60 °C was required. DGEBA was processed at ambient conditions. The mold was treated with a release agent in order to prevent the foam from sticking to the walls. For each type of resin, the specific amount of resin–hardener mixture was prepared in order to ensure a proper filling without overmolding. 

The corresponding amounts of hardener (mixtures if specified) and resin (blends if specified) were added in a 10-mL speed mixer cup and mixed at room temperature for 1 min at 3500 rpm. It is noted that when using the hardener mixture or TSH, the blowing agent is mixed into the resin first, followed by a second mixing step where the AEP was added. Agglomerated B-AEP or TSH particles were gently pounded with a wooden spatula before the resin was added.

The epoxy and hardener mixtures, with respective amounts listed in Table 1, were casted into the open mold. The mold was closed and transferred into a circulating air oven at 140 °C for 30 min for the foaming and curing process. A cooling step was carried out using two metal plates (150 × 150 × 30 mm) for 5 min. After that, the mold was opened, the epoxy foam was released, and finally, it was left at room temperature in the fume hood.

An overview of the prepared and investigated samples is given in Table 1.

#### 2.2.3. Characterization

Differential scanning calorimetry (DSC) was used to determine reaction kinetics and glass transition temperature (*T_g_*). Therefore, a DSC1 Stare System (Mettler Toledo, Columbus, OH, USA) was used. All the measurements were carried out with N_2_ at 50 mL/min. Dynamic measurements were proceeded with a heating rate of 10 °C/min and a heating cycle from −30 °C to 300 °C with a subsequent cooling to 25 °C followed by a second heating to 300 °C. First, the heating ramp provided the reaction onset, reaction enthalpy, and total reaction energy for the decomposition of the blowing agents, as well as the curing of the resin systems. The second heating provided the glass transition temperature, *T_g_*. 

Thermogravimetric analysis (TGA) was used to investigate the decomposition behavior of the blowing agents. The measurements were conducted with a TGA/SDTA851e (Mettler Toledo, Columbus, OH, USA). A heating ramp was set from 25 °C to 300 °C with a heating rate of 10 °C/min and 50 mL/min nitrogen as the carrier gas. 

In order to determine the viscosity profile during curing and the gel point, rheological investigations were conducted with MCR301 rheometer (Anton Paar, Graz, Austria) equipped with a 25-mm plate. Dynamic measurements were carried out from 25 °C to 150 °C at a heating rate of 2 °C/min with ω = 1 rad/s and γ = 10%. Isothermal measurements were carried out with ω = 1 rad/s and γ = 10%, and temperatures were chosen depending on the system. The gel points were determined by the overlap of storage modulus and loss modulus. The measurements were further used to understand the temperature dependence of the resin viscosity and the curing behavior. For the optical characterization of the cell structure, a DEM6000 (Leica, Wetzlar, Germany) light microscope was used. Complementary SEM analysis was made with a JSM-6510 (Jeol, Akishima, Tokyo, Japan). Samples were sputtered with a 13-nm gold layer by using a Sputter Coater 108auto (Cressington, Waltford, England). The gold used had 99.9% purity. For density measurements, sample cubes with a diameter of 1 cm³ were prepared out of the center area of the foamed samples with a Diadisc 6200 saw (Mutronic, Rieden am Forggensee, Germany) mounted with a diamond razor blade, in order to minimize the influence of a possible foam skin (skin was removed at each side, except from the top and bottom side). The density was measured by the Archimedes principle, where measurements were performed in distilled water with an AG245 analytical balance (Mettler Toledo, Columbus, OH, USA) with the use of a density kit for AG balances.

## 3. Results and Discussion

### 3.1. Pre-Foaming Experiments

#### 3.1.1. Unblocking Behavior of B-AEP and Decomposition Behavior of B-AEP and TSH 

**Figure 1:**
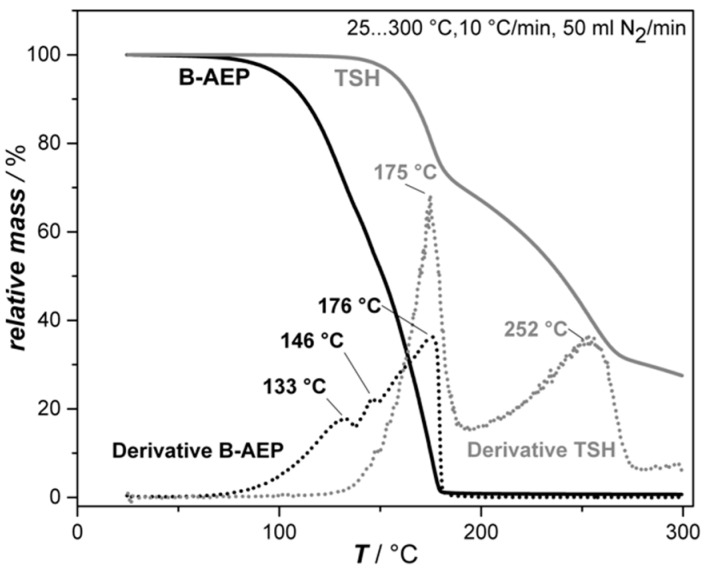
Thermogravimetric analysis (TGA) decomposition curves of B-AEP and TSH.

**Figure 2:**
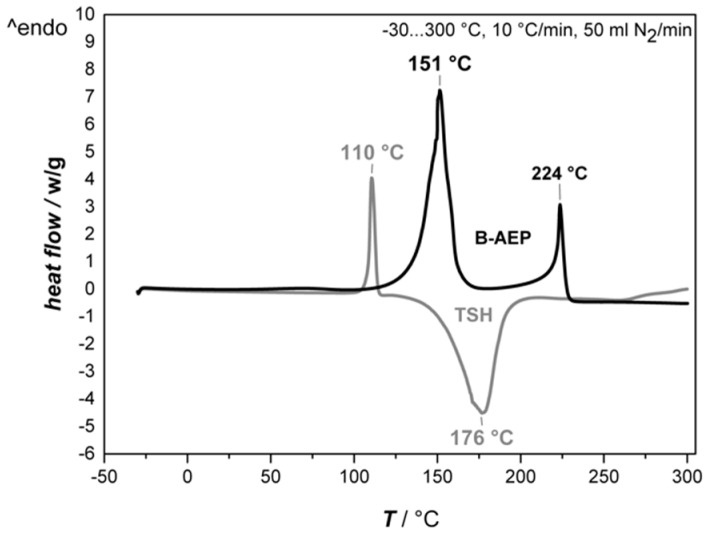
Differential scanning calorimetry (DSC) first heating curves of B-AEP and TSH.

In order to understand the unblocking behavior of B-AEP and decomposition behavior of TSH for the later foaming procedure, TGA and DSC measurements were carried out. The TGA curves of both foaming agents and their respective derivatives are compared in Figure 1, while their DSC first heating curves are shown in Figure 2.

The TGA analysis revealed that B-AEP (black lines) presents three different decomposition steps at different temperatures. The first decomposition step, onset with 1 wt.% loss at 77 °C, reaches its maximum at 133 °C, indicating the release of CO_2_ with a loss of 30 wt.%. The onset of decomposition can be related to the curing onset, as free AEP is available in the system at this time. The second peak at 146 °C indicates the release of further CO_2_ and water from the carbamate structure. Finally, the third step at 176 °C indicates mainly the volatilization of AEP. However, it is suggested that all peaks involve CO_2_ and water release, as well as the volatilization of AEP simultaneously in different amounts, as it is not possible to separate all the degradation steps independently [4]. Supplementary isothermal TGA measurements for B-AEP at 120 °C and 140 °C allowed estimating the time range of CO_2_ release. The total decomposition at 140 °C was found to be nearly twice as fast compared to 120 °C. The first step of decomposition corresponds to around 33% weight loss (about 1.4 mole CO_2_ per B-AEP). This peak was reached for 120 °C at 345 s and for 140 °C at 231 s. At these times, the major amount of CO_2_ is released. These experiments are relevant for the later foaming and curing experiments in order to adjust the proper time and temperature ranges of the process. The observed total volatilization in TGA of around 176 °C was found in multiple measurements and contributed to the measurement setup.

Within the DSC measurements, the decomposition of B-AEP showed two endothermal peaks. The first peak at 151 °C results in an enthalpy of 637 J/g. Here, the foaming relevant CO_2_ is released. The second peak at 224 °C with an enthalpy of 218 J/g indicates the final volatilization of AEP, as previously indicated by DSC investigations of AEP during pre-trials with an enthalpy of 285 J/g. This result matches with the information given in the material safety data sheet [25]. 

On the other hand, TSH exhibits a sharp endothermal peak at 110 °C with an enthalpy of 100 J/g, followed by a broad exothermal peak at 176 °C with an enthalpy of −556 J/g. The endothermal peak can be seen as ignition energy that is required to initiate the exothermal decomposition. In this case, the foaming relevant N_2_ and H_2_O are released, which is also observed within the TGA results, with a loss of approximately 70 wt.%. The second step observed in TGA indicates further decomposition, where sulfuric by-products are released (which are notable by odor).

#### 3.1.2. Effect of Blocked and Unblocked Blowing Agent on the Curing Enthalpy and Thermal Properties Relevant for the Foaming Process

The main difference in the decomposition enthalpy of the two blowing agents (endo versus exothermal) is important for later curing and foaming experiments. Therefore, the curing and foaming reaction was also investigated by DSC with a heating ramp of −30 °C to 300 °C at 10 °C/min 50 mL N_2_/min. The resulting values for the curing enthalpy ∆H for the different systems are given in Table 2.

The main reaction peak is shifted to higher temperatures for all the resin systems when B-AEP is used compared to AEP. This finding confirms the deactivation of the hardening functionality at B-AEP at lower temperature ranges, as its decomposition temperature is around 120 °C. Additionally, the energy consumption of the endothermal decomposition is shifting the main reaction peak to higher temperatures. When the hardener mixture is used, the main reaction peak temperature is lowered, as the exothermal curing reaction of AEP provides further energy, accelerating the decomposition of B-AEP. This effect can be seen best by comparing the DGEBA systems, where also a 50:50 AEP:B-AEP mixture was investigated. The peak at b-DGEBA (B-AEP only) presents the highest value of 133 °C, followed by m_1_-DGEBA (20% AEP) with 128 °C and m_2_-DGEBA (50% AEP) with 100 °C. Similar trends can be observed for the resin blend and EN system. The TSH systems present the lowest reaction peak temperature value, due to the exothermal decomposition of TSH, which is initiated by the curing reaction and speeds up the reaction at low measuring temperatures. The separation into two peaks when B-AEP is involved might be contributed to the reaction of primary and secondary amines, which are now separated due to the prior decomposition step. The AEP/B-AEP mixed systems show also two peaks, whereas the first peak is broadened, and shows an earlier onset caused by the free AEP content.

It is also observed that the use of B-AEP is increasing the curing enthalpy (less negative value) compared to systems containing AEP and systems containing AEP+TSH. Also, TSH decomposes exothermally; the ∆H value of non-foamed systems is still lower (higher negative value). Here, the foam morphology has to be taken into account, in which in situ changes the heat conductivity and sample–crucible interaction. This influence on the measurement has to be in mind regarding all foamed systems. Comparing the foam samples only, a significant influence of AEP+TSH, namely more than −100 J/g compared to B-AEP systems, can be observed. Within the samples containing hardener mixtures, a slight decrease of ∆H can be observed because of the substitution of B-AEP by AEP. These results helped, combined with pre-trials, to define the curing parameters for all systems in order to minimize an overheating effect within the samples.

#### 3.1.3. Rheological Behavior of Blending of DGEBA and EN and their Blend 

**Figure 3:**
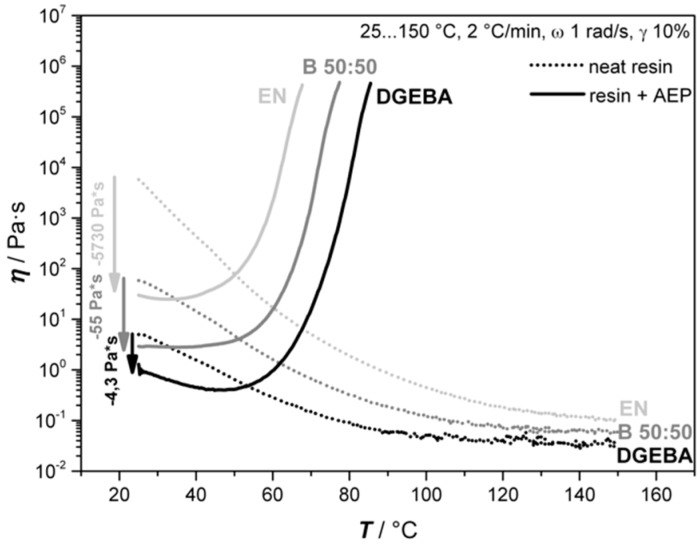
Rheological behaviors of DGEBA, EN, and their 50:50 blend (B 50:50) as neat resins and as a mixture with a stoichiometric amount of AEP.

**Figure 4:**
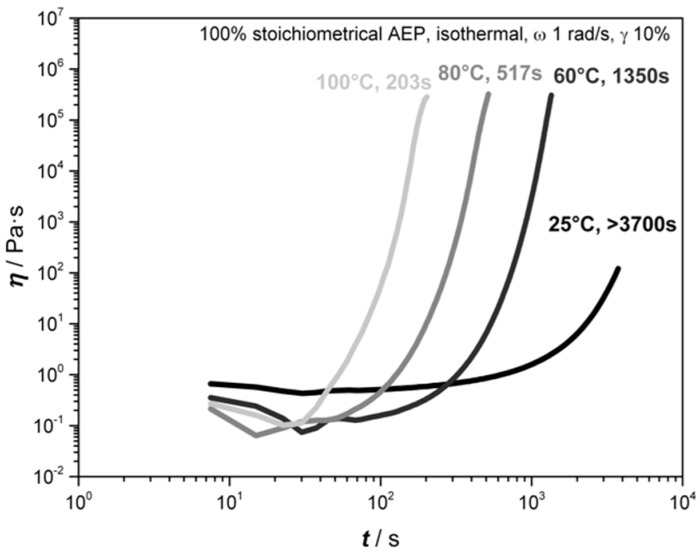
Rheological investigations of DGEBA with a stoichiometric amount of AEP at different isothermal temperatures.

These experiments address the rheological properties of the resins DGEBA, EN, and its 50:50 blend system with regard to their curing behavior when AEP is used as a hardener, as well as its thermal properties. Other mixing ratios were investigated during pre-trials, and are not presented here due to the direct correlation of the mixing ratios of DGEBA and EN, which are here represented by the 50:50 blend. The investigations are of importance, as the time scale before reaching the gel point has to be identified to be sufficient in order to allow the expansion and stabilization of the bubbles before coalescence or collapse. Figure 3 presents the results of the rheological investigations of the three resin systems without and with 100% stoichiometric AEP, while Figure 4 illustrates the isothermal measurements of DGEBA+AEP at different temperatures and the corresponding gel point.

It is observed from Figure 3 that the liquid nature of AEP leads to a decrease in viscosity at the beginning of the measurement compared to the neat resin systems. As expected, the lowest viscosity was observed at the DGEBA system, with an increasing value when EN is added. Moreover, it was observed that when using 100% stoichiometric hardener, the gel point is firstly reached by the EN system at 68 °C (21 min), followed by the 50:50 mixture at 77 °C (25 min) and finally by the DGEBA system at 85 °C (30 min). The shape of the graphs shows the typical three regions of a curing resin. At the beginning, the increase in temperature leads to a drop down in viscosity as the resins begin to soften (seen also at neat resins). This is followed by a change in slope as the hardener turns active. This change can be either smooth, leading to a plateau-like region as seen by the blend 50:50 curve, or stronger, leading to a curve-like shape. The nature of the change depends on the curing kinetics and interaction of the oligomers. Finally, the third region describes the active phase of the hardener where the viscosity increases continuously. The molecular structure plays also an important role regarding the gel point, as the bifunctional DGEBA owns a different oligomer formation compared to EN, which has a functionality around 3.6 [23].

Subsequently, the curing behavior dependence on temperature was investigated thorough rheological measurements of DGEBA+AEP with isothermal conditions at 25 °C, 60 °C, 80 °C, and 100 °C (Figure 4). It can be seen that by increasing the temperature, the reaction time diminishes; thus, the gel time decreases. At 25 °C, the gel point is obtained after 3600 s, at 60 °C, it is reached at 1350 s, while at 80 °C, it is observed at 517 s and at 100 °C, it takes about 174 s. However, it has to be taken into account that the gel point does not indicate the end of chemical reactions. Due to the decomposition temperature of B-AEP being between 100–120 °C, the gel point indicates a processing window of 174 s where foaming and curing takes place when AEP is released. The decomposition kinetics of the B-AEP leads to a delay of the gel-point, as it was observed to be 345 s until all CO_2_ is released from TGA. In a foaming scenario, the speed is also depending on the heating rate and heat diffusion given in the oven and mold.

**Figure 5:**
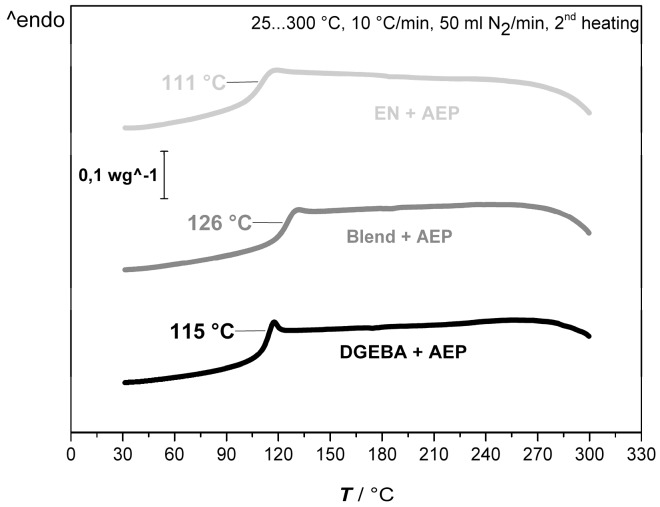
Second DSC heating curve of DGEBA, 50:50 blend, and EN resin systems with 100% stoichiometric AEP for the determination of *T_g_* as indicated by the change of the slope.

The observed direct influence of the blending leads to further experimentation with neat DGEBA, EN, and its 50:50 blend only, as the compatibility between resins and possible effects of the blending in the foaming procedure can be investigated in a sufficient way. Figure 5 compares the *T_g_* of the DGEBA, EN, and its 50:50 blend resin system cured with AEP received from the second heating curve from DSC.

The overall glass transition behavior of all the systems is in a close range. The determination of the *T_g_*, by its definition as the change in the slope, leads to the following observation: the lowest *T_g_* can be observed by EN (111 °C) followed by DGEBA (115 °C). The increased *T_g_* and that only one *T_g_* can be observed at the 50:50 blend (126 °C) is indicating a proper miscibility of the two resins, which result in a better performance [26]. Within this study, the *T_g_* was determined by the second heating curve of the DSC measurement in every case. This ensured a comparability of every measurement. It has to be kept in mind that further post-curing of the resin systems might lead to higher *T_g_* values. This step was not further investigated, as it was not the aim of this work.

#### 3.1.4. Hardener Mixture 

During pre-trials, it was observed that a high amount of CO_2_ was released before the viscosity was sufficient to enable a foam expansion without cell wall breakage. Therefore, the idea of mixing a blocked and unblocked hardener could provide a solution to this problematic by increasing the viscosity thorough a precuring step before the decomposition of B-AEP takes place, in order to minimize the loss of CO_2_ when the blocked hardener turns active and finally cures the complete system above its gel point.

**Figure 6:**
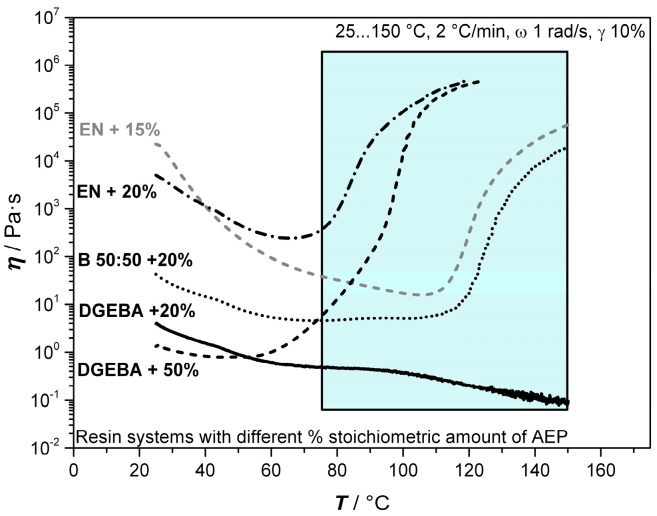
Rheological investigations of DGEBA, EN, and its 50:50 blend with a stoichiometric amount of AEP (presented in % amount relative to 100% stoichiometric). The grey region illustrates the range, where B-AEP decomposition is releasing further AEP in the foaming process, whereas a different viscosity behavior is expected.

The amount of AEP required for precuring was determined by dynamical rheological investigations of the three chosen resin blends and different amounts of AEP, namely 10%, 20%, 50%, and 100%. In the case of EN, 15% were also investigated, as 20% showed a higher curing activity as expected, while 10% was too far from the chosen 20% content of the other systems. Figure 6 exhibits the results for the formulations presented in this work.

As expected, the results indicated that by lowering the AEP content, a higher viscosity is observed. When using 20% AEP content, DGEBA shows little influence, as only a slight change in slope, around 60 °C, was observed. The heat-induced lowering of viscosity by around 100 °C will be overcome later during foaming, as B-AEP decomposition starts around 77 °C. In contrast, the EN system containing 20% shows a strong increase in viscosity, around 70 °C, and reaches its gel point after 2860 s around 120 °C. The use of 15% AEP at EN presents a different behavior. In this case, a first change in slope can be observed around 50 °C, with a further decrease in viscosity. Around 110 °C, the crosslinking begins and overcomes the thermal softening, which is observed by an increase of viscosity. However, the system does not reach its gel point within the 4500-s experimental time. These observations at EN lead to the conclusion that 20% AEP is not beneficial for foaming experiments due the fast gelation. An amount of 15% leads to a sufficient increase of the time window for curing and foaming. It has to be kept in mind that during the decomposition of B-AEP, further AEP is released into the system, leading to a shift of the gel point to lower time scales. Due to the gas release, no rheological investigations containing B-AEP could proceed. The 50:50 blend system shows a decrease in viscosity at the beginning, followed by a plateau-like state around 70 °C, which is similar to the DGEBA system. The overall higher viscosity can be contributed to the EN content, which also leads to a significant increase of viscosity around 115 °C. This tendency is due to the bifunctional structure of DGEBA, which only connects linearly to two AEP molecules, while EN allows reacting with up to four AEP molecules. Thus, EN leads faster to bigger oligomers that contribute to higher viscosities. The additionally increased crosslinking of EN also promotes a faster increase in viscosity, even at low amounts of AEP. The 50:50 blend system also does not reach its gel point during the 4500-s experimental time. DGEBA was additionally investigated for a 50:50 blocked–unblocked hardener mixture. This system shows a change in slope twice, around 60 °C and 120 °C. This effect can contribute to the two reaction steps of the primary and secondary amines. The primary amines react to linear oligomeric structures with DGEBA, while the secondary amines react to branches and finally networking structures, as observed by the change in slope. This time, the viscosity is increasing significantly and reaches the gel point around 124 °C (2970 s).

Pre-investigations with model-free kinetics (MFK) on an AEP+Blend 50:50 system allowed to support the timeline findings of the rheological investigations by a kinetic model related to the AEP amount. According to the rheological results of DGEBA at 60 °C, the gel point occurs at around 22.5 min, corresponding to a conversion of 77% at the MFK. While at 80 °C, the gel point can be observed after 8.6 min, which corresponds to a 75% conversion according to the MFK. These results verify a general conversion of around 76% before the system reaches its gel point. This correlation between the gel time and specific conversion can be a promising tool for time-saving process planning, and confirms the need of post-curing in order to achieve high conversions in the products.

### 3.2. Foaming Experiments

During these experiments, next to the differences in curing kinetics, the differences in viscosity are expected to influence the foaming behavior [27]. Against initial expectations, not all of the obtained foams show the formation of a notable foam skin. Only systems with unblocked hardeners tend to form a solid and smooth skin. As a reason, the contact of the unblocked hardener with the hot mold can be seen, as it is initiating the curing reaction already during the heating, before the blowing agents turn active. In case of the B-AEP systems, the curing kinetics with simultaneously in situ blowing and curing leads also to bubble formation and freezing in the outer regions, as the resin is not able to cure by touching the wall before B-AEP decomposes. Thus, structures of closed cells are observed at the outer regions. 

**Figure 7:**
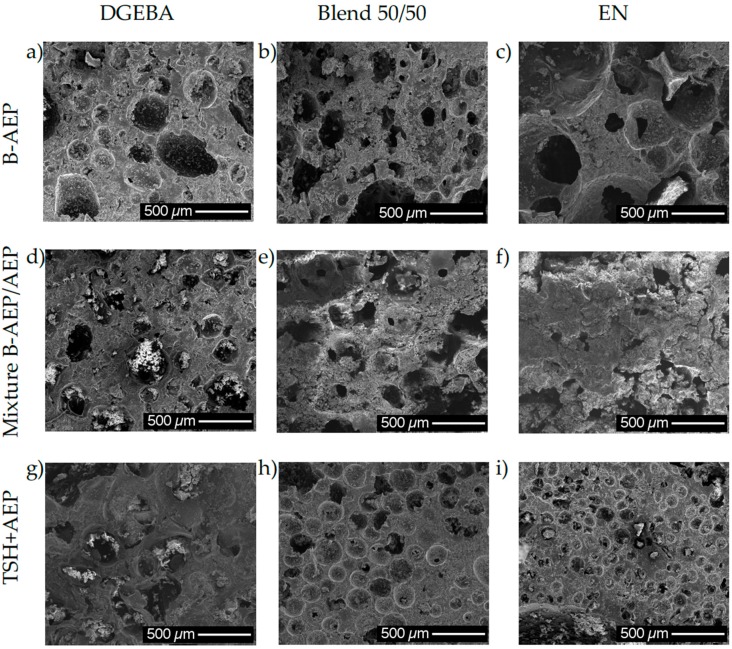
SEM images of central regions of foams obtained by different formulations.The foams obtained by the use of B-AEP only with DGEBA (**a**), blend 50/50 (**b**) and EN (**c**) do not contain any unblocked AEP. The B-AEP/AEP mixtures were 80/20 of the stoichiometric amount required for the curing at DGEBA (**d**) and the blend 50/50 system (**e**); and 85/15 at EN (**f**). The systems containing TSH as blowing agent with DGEBA (**g**), blend 50/50 (**h**) and EN (**i**) contain 100% stoichiometric ublocked AEP.

The core regions present inhomogeneous cell morphology and a broad range in cell size distribution. As a possible reason for the inhomogeneous cell distribution, the particle size distribution of the blowing agents is named in the literature [4], leading to inhomogeneous gas release and nucleation among the system. This is especially important for the systems containing AEP, as the released gas is entrapped faster due to the higher initial viscosity at decomposition temperature. As shown by MFK, the gel point is reached at around 76% conversion. Thus, even with B-AEP, a spontaneous locally higher gas release can be entrapped and form bigger bubbles compared to regions with lower gas release. An overview of the foam core regions is given in Figure 7.

From Figure 7, the expected influence of the viscosity and functionality of the resin system can be seen by comparing the resulting foams among the different blowing and hardening systems. As already shown in Figure 3, EN is able to increase the viscosity of the system faster compared to DGEBA. This effect leads to the general trend of the cell morphology to get more homogeneous with increasing content of EN resin, which can be seen from comparing image sets with B-AEP (Figure 7a–c), hardener mixture (Figure 7d–f) and TSH (Figure 7g–i). The cell size distribution is lowered. This is in accordance with common theories previously displayed in the literature [27]. Due to the inhomogeneous cell size distribution, no proper evaluation of cell size distribution is possible. Especially within the light microscopy, partially additional small bubbles can be observed within the cell struts, contributing to cells in a region <100 µm next to bubbles that can be obviously observed with the naked eye >5000 µm.

**Figure 8:**
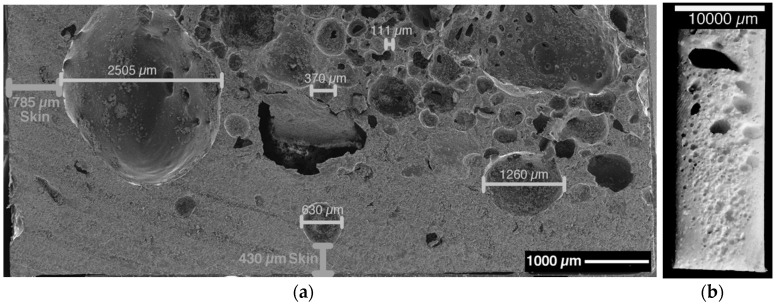
Integral foam structure of m2-DGEBA (50:50 stoichiometric AEP and B-AEP). (**a**) SEM image of top left edge region with some indicated values of skin thickness and cell diameter; (**b**) Photo of sample center region at sample cured vertically for better illustration of cell size distribution; bottom small sized, upwards increasing.

Additionally, the different blowing and hardening systems lead to different morphological structures among the different resin systems. As explained, especially the mixtures containing AEP own two-step kinetics, whereas the system begins already to cure upon heating before the blowing agent turns active. This effect leads to the formation of a foam skin on the samples containing a B-AEP/AEP hardener mixture or AEP only with TSH. This was tested to an extreme with a 50/50 of stoichiometric amount B-AEP/AEP mixture for DGEBA. In this case, a clear, structural foam structure with a foamed core and solid skin can be observed among the samples, as presented in Figure 8.

The foam skin is around the whole sample with at least 400-µm thickness, depending on the sample position within the mold. The inhomogeneous cell size distribution in the foam core can be observed within Figure 8. Additionally, a gradient in cell size was observed from the bottom to the top side. Due to the upwards movement of the gas, coalescence effects in the top region lead to the formation of bigger bubbles compared to the bottom region, where especially at the edges, only little amounts of cells are located. Under given experimental conditions, the TSH samples especially present the most inhomogeneous cell sizes and a broad distribution. In this case, multiple aspects have to be considered. Next to the dispersion quality, the blowing agent does not match to the curing kinetics of the hardener under given conditions. In general, the TSH systems showed the highest sensitivity during processing, as the blowing agent began to decompose before proper mold filling under certain conditions. In this case, the exothermal curing reaction initiates with the pre-heated mold, leading to an even faster curing and decomposition of more TSH at one time. This spontaneous decomposition of large amounts in a short time can be seen as the reason for big bubbles in the systems. 

**Figure 9:**
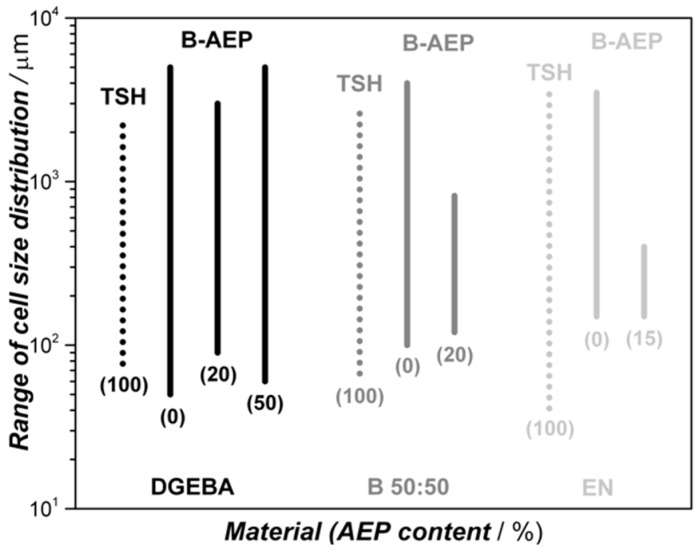
Range of cell size distribution (left side) among the different foam samples by resin and AEP content (percentage of AEP in brackets below the lines). Dotted lines represent the use of only TSH as the blowing agent. Shorter lines illustrate a smaller cell size distribution.

Figure 9 qualitatively illustrates the evaluated cell size distributions found via light microscopy and SEM analysis, they are also confirmed by naked eye impressions of cell morphology (see Figure 8).

The range of determined cell sizes can be compared among the different systems, where certain tendencies can be observed. The influence on foaming behavior by the viscosity is found to be confirmed according to literature [27] for the systems containing B-AEP. The cell sizes are getting more homogeneous by increasing the EN content, and thus viscosity, in the systems. The effect of adding an unblocked hardener increases the viscosity before the foaming step, resulting in a smaller cell size distribution. At the 50/50 AEP/B-AEP system, the increase compared to the 20/80 AEP/B-AEP system can be explained by the formation of the integral foam structure, leading to an increased gas load in the foamed core region that supports inhomogeneous gas distribution with spots of high and low gas load. This effect can also be observed at the TSH foamed samples (Figure 9, dotted lines), where agglomerated TSH and the less controllable curing kinetics lead to a broad cell size distribution. In this case, the high viscosity EN samples present smaller cells, especially close to the edges, as the coalescence cannot take place due to fast curing near the mold wall. In contrast, the center region is expected to be low viscosity at this point in time, due to the heat release of the exothermal reaction enthalpy and TSH decomposition, leading to coalescence. The foams with the best performance regarding cell morphology are the blend 50:50 system containing the 20/80 AEP/B-AEP mixture and the EN system containing the 15/85 AEP/B-AEP mixture, as respectively seen by shortest lines in Figure 9. Within these two systems, the viscosity, decomposition, and curing kinetics were synchronized to an optimum in foam morphology under given experimental conditions.

**Figure 10:**
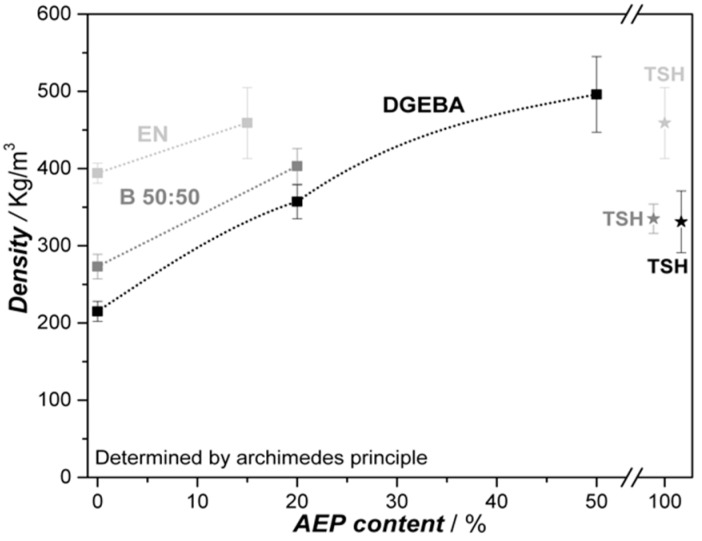
Foam density of different resin samples in dependency of AEP content, 0% means use of B-AEP only, and the TSH benchmark, where 100% AEP is required.

Regarding the resulting foam density, the influence of the different resins can be seen as the governing factor, where the blowing systems are subordinated. Figure 10 presents the foam densities determined by the Archimedes principle for each system.

All the systems under investigation are able to decrease the density more than 50% compared to the compact resins cured with AEP (density DGEBA 1160, blend 50:50 1180, EN 1200 kg/m^3^). The lowest densities are achieved with DGEBA resin, independent from the blowing system. Due to the high inhomogeneity of the TSH foams, the samples of DGEBA and the 50:50 blend resin are in a close range. The network density of DGEBA is lower compared to EN, as the functionality of EN is 3.6, while DGEBA owns a functionality of two. Thus, the expansion ratio of both resins is different, which is most notable during foaming pre-experiments and the sample preparation, where only 70 wt.% of the DGEBA sample was required compared to EN in order to achieve a properly foam-filled mold (see Table 1). On one hand, a decrease of B-AEP content in the mixtures is logically expected to lead to an increase in density, as the amount of CO_2_ is decreasing. On the other hand, the observed CO_2_ loss during pre-trials lead to the expectation that a substitution of an amount of B-AEP by unblocked AEP could equal the CO_2_ loss by means of increasing the viscosity before its decomposition. The experiments revealed that the setup was not suitable to figure out the precuring effect at the expected level, as the heat transfer in the mold was insufficient and the AEP lead to the formation of a foam skin, as it was not sufficiently heated all through the sample. Nonetheless, the cell size distribution was lowered by the hardener mixtures, and the overall cell morphology improved. At this point, a further understanding of the correlation between curing kinetics and the activity of the blowing agent has to be achieved for low densities combined with proper cell morphology. Further investigations regarding this aspect are in progress.

## 4. Conclusions

Two different epoxy resins as well as its 50:50 blend were foamed by the use of the CO_2_ blocked hardener AEP, which is named B-AEP, and was synthesized by the authors. This carbamate hardener is able to release CO_2_ in situ during the process, leading to a simultaneous curing and foaming. The pure resin+B-AEP system was found to be less sensitive to processing fluctuations, especially regarding temperature issues upon heating, compared to the benchmarked TSH and AEP/B-AEP mixed systems. This processing stability can be contributed to the carbamate salt structure of B-AEP. Additionally, the pure B-AEP systems showed the lowest tendency to form a foam skin during closed mold batch foaming with all three resin systems. The different amounts of unblocked AEP, as well as the choice of resin, influence the morphology as a reason for the change in viscosity, functionality, and curing kinetics. Hence, next to additives, the resin itself was identified as a striking influencing factor regarding the cell morphology. The cell size distribution is lowered by increasing EN resin content; however, the density is increased. Here, the higher viscosity, network density, and curing kinetics can be seen as governing factors for both effects. As expected, the different curing and blowing systems (B-AEP, AEP+B-AEP, or AEP+TSH) do not affect the *T_g_,* as it is depending on the type of resin. The best performance regarding foam morphology and cell size distribution was found for two systems: the AEP/B-AEP mixtures combined with the 50:50 blend and the EN resin.

The basic knowledge generated within this work can be seen as a further step for new foaming systems with environmental-friendly CO_2_ as the latent blowing agent.

## Figures and Tables

**Table 1:** Overview of formulations prepared for foaming (% is related to stoichiometric hardener equivalent), the amount of mixture required for proper mold filling of expanded foam, and the final density achieved with respective formulation. AEP: N–aminoethylpiperazine, B-AEP: blocked hardener N-Aminoethylpiperazine, DGEBA: diglycidyl ether of bisphenol-A, EN: epoxy-novolac, TSH: toluenesulfonyl hydrazide.

Formulation	Resin	Hardener	Blocked Hardener	Blowing Agent	Amount [g]	Density [kg/m^3^]
b-DGEBA	DGEBA	B-AEP			3.2	215 ± 13
m1-DGEBA	DGEBA	AEP (20%)	B-AEP (80%)		4.0	357 ± 22
m2-DGEBA	DGEBA	AEP (50%)	B-AEP (50%)		5.1	496 ± 49
DGEBA-TSH	DGEBA	AEP (100%)	-	TSH (10 wt.%)	3.5	331 ± 40
b-Blend	DGEBA:EN 50:50	B-AEP			3.2	273 ± 16
m1-Blend	DGEBA:EN 50:50	AEP (20%)	B-AEP (80%)		4.0	403 ± 23
Blend-TSH	DGEBA:EN 50:50	AEP (100%)	-	TSH (10 wt.%)	3.6	335 ± 19
b-EN	EN	B-AEP			3.5	357 ± 43
m3-EN	EN	AEP (15%)	B-AEP (85%)		4.0	394 ± 13
EN-TSH	EN	AEP (100%)	-	TSH (10 wt.%)	4.4	459 ± 46

**Table 2:** Overview of samples and its corresponding temperature of highest reaction (peak), the curing enthalpy ∆H, and the *T_g_* (second heating) determined by DSC with a heating ramp of −30...300 °C at 10 °C/min and 50 mL N_2_/min.

Formulation	Peak [°C]	∆H (J/g)	*T_g_* [°C]
AEP + DGEBA	93	−501	115
b-DGEBA	133/155	−208	104
m_1_-DGEBA	128/160	−238	99
m_2_-DGEBA	100/160	−282	106
DGEBA-TSH	84	−375	98
AEP + Blend 50:50	90	−482	126
b-Blend	132/160	−127	117
m_1_-Blend	124/158	−168	106
Blend-TSH	82	−403	107
AEP + EN	86	−417	111
b-EN	130/164	−200	121
m_3_-EN	118/155	−201	121
EN-TSH	77	−334	121

**Figure A1:**
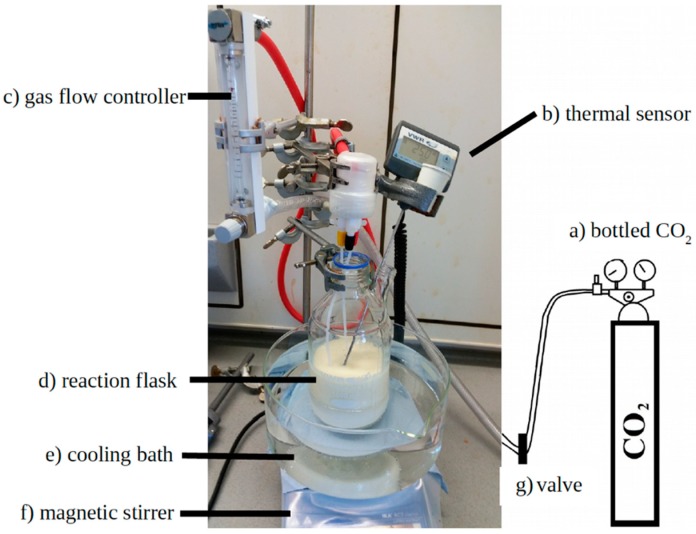
Experimental setup for B-AEP synthesis.

**Figure A2:**
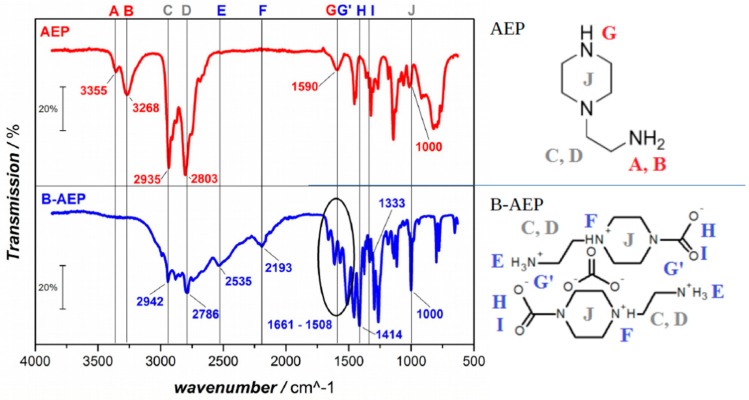
Fourier transform infrared (FTIR) spectra of AEP and B-AEP; the lines and wave numbers show significant peaks and/or missing peaks according to the literature [4].

## Appendix A

### B-AEP Synthesis and Characterization

The samples were prepared by mixing EtOH and AEP in a ratio 5:1 (about 390 g of EtOH, technical grade and 80 g of AEP). After mixing by gently shaking the flask for 30 s, the reaction flask was attached to the experimental setup (Figure A1), and the cooling bath was attached. The bottled CO_2_ (grade 4.5) was bubbled with a flow rate of 100 mL/min into the solution for 360 min. Despite all precautions, the flow rate showed some fluctuations over time, which did not influence the final product quality. After the reaction time, the product was filtered and washed with 300 mL of EtOH. The received powder was dried in the fume hood overnight and set into the vacuum chamber at 100 mbar and 40 °C for 16 h. A total amount of 91 g was received after drying. The dried samples were investigated by FTIR (Figure A2) in order to confirm the carbamate formation according to the literature [4].
